# Effects of Temporal Processing on Speech-in-Noise Perception in Middle-Aged Adults

**DOI:** 10.3390/biology13060371

**Published:** 2024-05-23

**Authors:** Kailyn A. McFarlane, Jason Tait Sanchez

**Affiliations:** 1 Roxelyn and Richard Pepper Department of Communication Sciences and Disorders, Northwestern University, Evanston, IL 60208, USA; jason.sanchez@northwestern.edu; 2 Knowles Hearing Center, Northwestern University, Evanston, IL 60208, USA; 3 Department of Neurobiology, Northwestern University, Evanston, IL 60208, USA

**Keywords:** speech-in-noise perception, temporal processing, temporal fine structure, pitch encoding, cochlear synaptopathy, middle-aged adults

## Abstract

**Simple Summary:**

A significant portion of adults with clinically normal hearing sensitivity have difficulty understanding speech in background noise. Current clinical assessments fail to explain this phenomenon, prompting the exploration of auditory mechanisms beyond those covered by routine clinical testing. One mechanism important for separating sound sources—a key task for understanding speech-in-noise—is temporal processing, or the extraction and organization of acoustic timing characteristics. Here, we investigate the hypothesis that deficits in temporal processing contribute to difficulties in understanding speech-in-noise. We explore this in middle-aged adults—an under-investigated group, despite their high prevalence of speech-in-noise difficulties. In this study, we found that differences in speech-in-noise abilities were associated with deficits in two aspects of temporal processing: the neural encoding of periodic speech features, such as pitch, and perceptual sensitivity to rapid acoustic timing differences between ears. Interestingly, the use of these mechanisms was task-dependent, suggesting various aspects of temporal processing differentially contribute to speech-in-noise perception based on the characteristics of the listening environment. These findings contribute to our overall understanding of which auditory mechanisms play a role in speech-in-noise difficulties in normal hearing listeners, and can inform future clinical practice to serve this population.

**Abstract:**

Auditory temporal processing is a vital component of auditory stream segregation, or the process in which complex sounds are separated and organized into perceptually meaningful objects. Temporal processing can degrade prior to hearing loss, and is suggested to be a contributing factor to difficulties with speech-in-noise perception in normal-hearing listeners. The current study tested this hypothesis in middle-aged adults—an under-investigated cohort, despite being the age group where speech-in-noise difficulties are first reported. In 76 participants, three mechanisms of temporal processing were measured: peripheral auditory nerve function using electrocochleography, subcortical encoding of periodic speech cues (i.e., fundamental frequency; F0) using the frequency following response, and binaural sensitivity to temporal fine structure (TFS) using a dichotic frequency modulation detection task. Two measures of speech-in-noise perception were administered to explore how contributions of temporal processing may be mediated by different sensory demands present in the speech perception task. This study supported the hypothesis that temporal coding deficits contribute to speech-in-noise difficulties in middle-aged listeners. Poorer speech-in-noise perception was associated with weaker subcortical F0 encoding and binaural TFS sensitivity, but in different contexts, highlighting that diverse aspects of temporal processing are differentially utilized based on speech-in-noise task characteristics.

## 1. Introduction

Difficulty understanding speech-in-noise, despite having clinically defined normal hearing, is a longstanding puzzle in auditory research and hearing health care. An estimated 26 million adults in the United States alone experience this phenomenon [1], making up about 10% of an audiologist’s caseload [2]. Yet, standard clinical audiometric evaluation often fails to validate or explain these patient complaints [3], leading to self-directed solutions (e.g., reducing social activity) that can negatively impact psychosocial well-being [4]. There is a critical need to identify measures for effective identification and management of this issue in clinical practice. To do so, we must first investigate the potential mechanisms contributing to speech-in-noise difficulty in adults with normal hearing sensitivity, particularly those not routinely evaluated in the clinic [5].

Auditory temporal processing is a vital component of auditory stream segregation, as it plays a role in forming perceptual features such as pitch and location [6,7]. Numerous studies have demonstrated a relationship between age-related deficits in temporal processing and decreased ability to understand speech-in-noise [8,9,10,11,12]. However, the role of temporal processing on speech-in-noise perception has been under-investigated in middle-aged listeners, where speech-in-noise difficulties are often first reported [2,13], and where confounds associated with older age (e.g., high-frequency hearing loss, cognitive decline) are less prevalent [14]. Therefore, the current study aimed to elucidate the role temporal processing plays in speech-in-noise perception for middle-aged listeners. Multiple temporal processing mechanisms within the auditory system were evaluated using electrophysiological and behavioral methods. A brief background for each of these mechanisms is provided in the following section, followed by an introduction to how speech-in-noise test characteristics may influence our findings.

### 1.1. Mechanisms of Temporal Processing Investigated

First, the auditory nerve is the initial site of neural encoding of temporal characteristics of sound [15]. Seminal work in animal models revealed that noise- and age-induced degeneration of the synaptic connections between the cochlear inner hair cells and auditory nerve fibers (commonly referred to as cochlear synaptopathy) can occur before permanent changes in hearing thresholds [16,17,18], spurring the idea that cochlear synaptopathy may be a hidden pathology in humans contributing to suprathreshold auditory deficits, such as difficulty understanding speech-in-noise [15,19]. A common indirect measure of the health of the cochlear synapses is the wave I peak-to-trough amplitude of the auditory brainstem response (synonymous with the compound action potential in electrocochleography), as it reflects the summed, synchronous neural activity at the peripheral auditory nerve. The synchronous firing of a population of auditory nerve fibers depends on the ability of neurons to consistently and precisely fire in time with the stimulus—a neural property known as phase-locking [20]. Neuronal loss or cochlear synaptopathy can result in dyssynchronous firing patterns (i.e., reduced phase-locking) [17], reflected in early auditory-evoked potentials as reduced peak-to-trough amplitudes [21]. Indeed, animal work has confirmed that the degree of synaptic damage is revealed in reduced wave I amplitudes [16,22], demonstrating a negative effect on temporal processing at the peripheral auditory nerve due to cochlear synaptopathy. Some human studies have demonstrated an association between reduced wave I amplitudes (i.e., degraded temporal coding) and speech-in-noise deficits [23,24,25,26], though the results across the literature are overall mixed [27,28]. Here, we evaluated temporal coding fidelity at the peripheral auditory nerve in our middle-aged cohort by measuring the change in wave I amplitude as a function of increasing stimulus repetition rate.

Second, differences in the subcortical encoding of periodic temporal features of speech have also been suggested to account for individual differences in speech-in-noise perception. Before speech signals reach the auditory cortex, their temporal information is encoded and preserved along the ascending auditory brainstem and integrated by the highest-order nuclei, the inferior colliculus [29]. Scalp-recorded evoked potentials originating largely from the inferior colliculus show precise, phase-locked neural activity to spectral and temporal features of speech, including features critical to pitch processing [29,30]. Pitch, a perceptual feature closely tied to a signal’s fundamental frequency (F0), is an important acoustic cue for identifying and separating target speech from competing signals [8,31,32]. The accurate representation of F0 relies on the precise coding of these temporal features by the central auditory system, beginning in the auditory brainstem [29,30]. Thus, changes to subcortical F0 encoding could negatively impact speech-in-noise perception, which has been shown to occur due to aging, noise exposure, and potentially auditory nerve degeneration [15,19,21]. Indeed, there is evidence that speech-in-noise perception is hindered in cases of weaker subcortical F0 encoding [8,31,33]. Here we measured subcortical F0 encoding in middle-aged listeners using the frequency-following response—an electrophysiologic measure of phase-locked activity to periodic features of sound originating in the brainstem [34,35]—to a consonant–vowel speech syllable.

Third, psychophysical studies have demonstrated that cues conveyed by an acoustic signal’s temporal fine structure (TFS) are important for understanding speech in background noise [7,36,37]. Information from TFS plays a role in the perception of pitch, sound localization, and the ability to detect a signal against a fluctuating masker [7,37]—all attributes critical to sound source segregation, such as discerning speech-in-noise. Indeed, perceptual measures of TFS sensitivity have repeatedly helped reveal an association between poor TFS sensitivity and deficits in speech-in-noise perception across various clinical populations (e.g., hearing loss and traumatic brain injury) [7,38,39], including aging adults with normal audiometric thresholds [10,40,41]. For this reason, we included a measure of binaural TFS sensitivity using a classic psychoacoustic task: frequency modulation (FM) detection [41,42]. A dichotic FM detection task was chosen over a monaural or diotic presentation, based on the literature supporting its ability to more acutely measure detection thresholds and identify emerging temporal processing deficits relatively early in the aging process [41].

Given the points described above, this study investigated the contribution of temporal processing to speech-in-noise perception in middle-aged listeners with normal hearing. First, we examined the strength of association between these three measures of temporal processing and speech-in-noise performance on a continuum. Next, we compared temporal processing abilities between this participant cohort’s top and bottom quartiles of speech-in-noise performers.

### 1.2. The Potential Influence of Speech-in-Noise Task Characteristics

While the primary research question of this study investigated the relationship between temporal processing and speech-in-noise perception in middle-aged adults, the study was designed to explore the possibility that this relationship may be more (or less) apparent based on the speech-in-noise test materials used. Described in detail by DiNino et al. [43], speech-in-noise tests vary in their sensory, perceptual, and cognitive demands based on their target and masker characteristics. For example, a speech-in-noise test with high-context sentences or stories allows listeners to rely on top-down (i.e., cognitive) resources, such as lexical and semantic knowledge, thus compensating for or overshadowing the presence of any sensory deficit (e.g., temporal processing deficits). Moreover, the degree to which the masker is perceptually similar to the target is likely to affect the reliance on sensory cues, such that higher similarity (i.e., more informational masking [44]) requires a larger reliance on the fine sensory cues critical to sound segregation [45].

As such, speech-in-noise tests that minimize top-down cues and maximize reliance on fine sensory details may more effectively demonstrate speech-in-noise difficulties due to sensory deficits. This notion was tested here by administering two speech-in-noise tests. First, the AzBio Sentence Lists [46] presented low-context sentences against a continuous 10-talker babble. While the target sentences have low predictability, individual differences in linguistic knowledge and vocabulary could influence performance and minimize the use of acoustic temporal cues. Additionally, the multi-talker babble reduces the perceptual similarity between the target and masker, potentially further minimizing reliance on temporal cues to segregate the target speech from the noise. Second, the spatial release from two talkers task (SR2) [47] presented sentences from a closed-set corpus spoken by three male talkers (one target, two maskers) that minimizes cognitive/linguistic resources and emphasizes the use of fine sensory cues (e.g., subtle differences in pitch) to segregate the target from the masker. Given the considerations summarized above, we expected to see stronger associations between measures of temporal processing and the SR2 task compared to the AzBio task. Interestingly, our findings instead show how diverse aspects of temporal processing are utilized differentially based on these factors.

## 2. Materials and Methods

### 2.1. Participants

**Figure A1:**
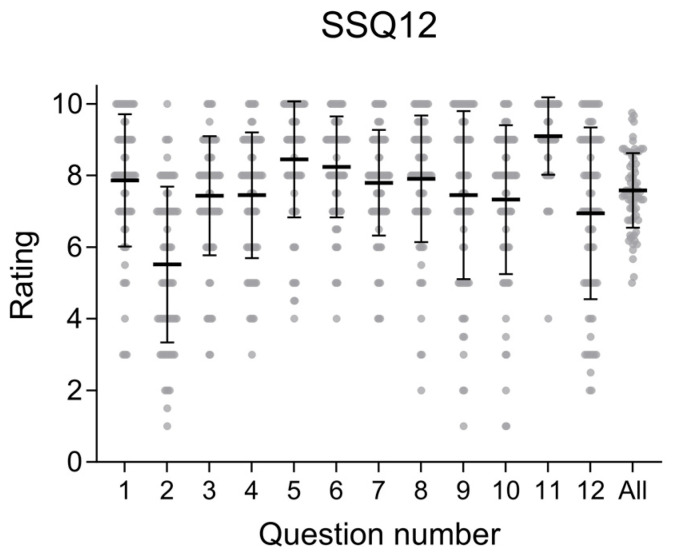
Distribution of self-rated hearing abilities across each SSQ12 question and the average score across all questions. A rating of 10 indicates no perceived difficulty in the given listening environment, while a rating of 0 indicates a perceived significant difficulty. SSQ12 questions can be found in Noble et al. [48].

Seventy-six volunteers (28 male) aged 30 to 50 (mean: 39.5 years, standard deviation: 6.16 years) participated in this study. Although there is a slight sex imbalance in our participant cohort, there is currently no consensus in the literature regarding sex-related differences in temporal processing in middle-aged adults. All participants were native English speakers. Before the data collection began, participants were informed of the experiment’s nature and we obtained consent. All experimental procedures were approved by the Northwestern University Institutional Review Board (STU00215893). Participants were not recruited based on a certain criterion of perceived speech-in-noise difficulty, as our research question concerned demonstrated listening abilities rather than perceived abilities. While not used for analysis, participants’ subjective ratings of their hearing abilities as measured by the SSQ12, a short form of the Speech, Spatial, and Qualities of Hearing scale [48], can be found in Figure A1. All data was collected over a single visit in a sound- and electrically-treated room in the Northwestern University Center for Audiology, Speech, Language, and Learning. The following methods are described in the same order in which they were administered.

**Figure 1:**
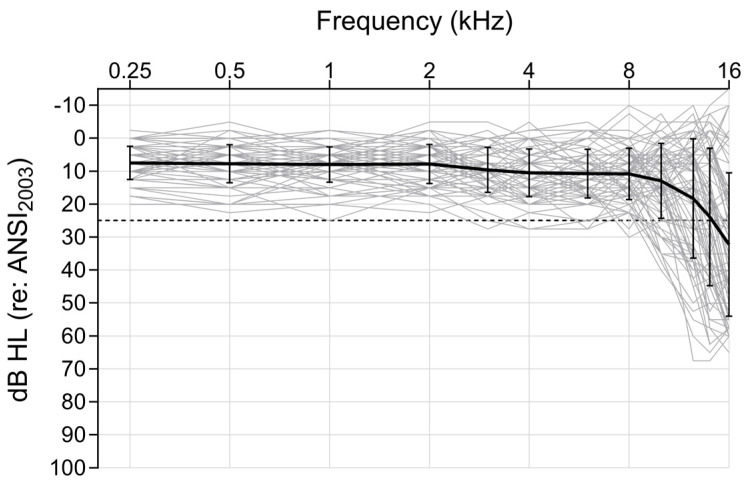
Audiograms of each participant (averaged across ears). Each participant is represented by an individual grey line. A mean audiogram (*n* = 76) is represented by the bolded black line, bracketed by ±1 standard deviation for each test frequency (0.25, 0.5, 1, 2, 3, 4, 6, 8, 10, 12.5, 14, 16 kHz). The dashed horizontal line indicates the cut−off for normal hearing (25 dB HL).

Hearing sensitivity was measured using pure-tone audiometry, performed in a sound-treated room using a GSI AudioStar Pro with Sennheiser HDA 200 circumaural headphones. Most participants had binaural thresholds within the range of normal hearing (≤25 dB HL) across the standard clinical test frequencies (0.25–8 kHz). Seven listeners had up to two thresholds outside the normal range (≤35 dB HL), but binaural thresholds within 30 dB HL were deemed acceptable by the authors. Participant thresholds at extended high frequencies (10, 12.5, 14, 16 kHz) were also collected. Non-response values (applied to four participants) for 14 and 16 kHz were set at 85 and 65 dB HL, respectively (i.e., 5 dB above the max output of the audiometer at that frequency). All participants had symmetrical hearing (e.g., no interaural threshold asymmetries ≥15 dB at more than one test frequency) through 8 kHz. Participants’ thresholds were averaged across ears at each test frequency for analysis. A 4-frequency pure-tone average (4-PTA; 0.5, 1, 2, 4 kHz) and an extended high-frequency average (EHF-PTA; 10, 12.5, 14, 16 kHz) were used in the analysis. Participant audiograms are presented in Figure 1.

### 2.2. Speech-in-Noise Testing

#### 2.2.1. AzBio Sentence Lists

The AzBio Sentence Lists (AzBio) presents conversationally produced, low-context sentences against a continuous 10-talker babble. Each AzBio list consists of 20 syntactically correct sentences (ranging from 3 to 12 words in a sentence), spoken by a rotation of two female and two male voices. The 10-talker babble comprises an equal number of male and female voices speaking syntactically correct sentences. The target and babble were presented at a 0 dB signal-to-noise ratio at 60 dBA through a loudspeaker positioned 3 feet in front of the seated participant at eye level. Each participant was administered one list from the ten available lists equal in intelligibility [49] via randomized selection.

Participants were instructed to listen to the sentences presented through the loudspeaker and repeat them verbatim, giving their best guess each time. Responses were recorded and scored as the percentage of correctly repeated words out of the total number of words presented across all 20 sentences. Only words from the target sentence were scored; any additional or alternative words in the participants’ responses were disregarded. Before the test condition, participants were familiarized with the task during a practice list presented at a +5 dB signal-to-noise ratio. Based on the lack of comparable normative data for AzBio performance, a relatively liberal criterion of three standard deviations was used to identify outliers. Two participants’ responses were excluded based on this criterion, resulting in 74 AzBio data points in the analysis.

#### 2.2.2. Spatial Release from Two Talkers

The spatial release from two talkers (SR2) task uses sentence-level stimuli from the Coordinate Response Measure Corpus [50], which takes the form “Ready [CALL SIGN] go to [COLOR] [NUMBER] now”. These sentences present eight call signs (Arrow, Baron, Charlie, Eagle, Hopper, Laker, Ringo, Tiger), four colors (blue, red, green, white), and eight numbers (1–8). Three male talkers simultaneously presented these sentences in co-located (all talkers at 0° azimuth) and spatially separated (maskers ± 45° azimuth) conditions. Participants were instructed to attend to the talker, who used the call sign “Charlie,” and select the color–number combination from a 32-element grid displaying all possible combinations of four colors and eight numbers. During testing, a progressive tracking procedure was used to find the target-to-masker ratio in dB, at which 50% accuracy was achieved. The primary outcome measure of the SR2 is a derived measure of spatial release from masking (SRM), calculated as the dB difference between target-to-masker ratios in the collocated and separated conditions.

The SR2 was administered on an iPad running the Portable Automated Rapid Testing application [51] with calibrated Sennheiser HD 280 Pro headphones. The target talker was presented at a fixed level of 65 dB SPL. Participants were seated at a desk with the iPad placed directly in front of them. The test was self-administered, with written instructions delivered within the application. Before the test trials, participants were familiarized with the response matrix during a practice block where the target “Charlie” sentences were presented without any distractor sentences. During testing, responses were collected via digital buttons on the iPad touchscreen (e.g., a grid of 32 color-number combinations). Correct/incorrect feedback was given following each response.

### 2.3. Psychoacoustic Testing

#### Dichotic Frequency Modulation (FM) Detection

To assess binaural TFS sensitivity, dichotic FM thresholds were estimated using a two-cue, two-alternative forced-choice task with adaptive tracking. This task was also administered on the Portable Automated Rapid Testing application immediately following the SR2 task. In short, this task presented four successive stimulus intervals in which either the second or third interval contained the target (i.e., modulated) stimulus, and the other intervals presented a standard (i.e., unmodulated) stimulus. Upon hearing all four intervals, the listener was instructed to choose whether the second or third interval contained the target. “Correct”/“incorrect” feedback was provided after each trial, and the degree of modulation of the target signal was adaptively adjusted based on the pattern of correct and incorrect responses using a two-stage, two-down, one-up procedure (i.e., increased difficulty after two correct responses, decreased difficulty after one incorrect response) [52].

The stimulus was a pure-tone carrier frequency randomly roving between 460 and 550 Hz across intervals. Each stimulus was presented at 75 dB SPL for 400 ms, followed by 250 ms of silence. The standard stimulus was presented diotically (identical at both ears). The target stimulus was a 2 Hz sinusoidal phase modulation that was inverted in phase between ears, creating a continuously shifting interaural phase difference determined by the modulation depth. The modulation depth was adjusted using an adaptive staircase algorithm on an exponential scale [52] until the listener’s detection threshold was estimated. Detection thresholds were log-transformed for analysis. Thresholds outside two standard deviations of normative data [38] were excluded, resulting in 74 dichotic FM data points in the analysis.

### 2.4. Electrophysiology

Electrode sites were prepped with alcohol and NUPrep™ skin prepping gel (Weaver and Company; Aurora, CO, USA). Surface electrodes (Ambu Neuroline surface electrodes; Ambu INC., Columbia, MD, USA) were placed onto the frontal midline (Fz), ipsilateral mastoid (M2), and contralateral mastoid (M1). A commercially available ear canal electrode (TIPtrode; Etymotic Research, Elk Grove Village, IL, USA) was placed in the ipsilateral ear canal (A2), ensuring the entire foam tip was inside the ear canal. For both electrophysiology measures, Fz served as the active/non-inverting electrode and M1 as the common ground. The TIPtrode was the reference/inverting electrode for electrocochleography recordings, and the M2 surface electrode was the reference for frequency following response recordings. Responses were successively acquired using the Intelligent Hearing Systems (IHS; Miami, FL, USA) SmartEP Duet platform (version 5.41.01).

Participants were seated comfortably in a reclined chair and prepped for electrode placement. Once all electrodes were placed, the impedance was confirmed to be ≤5 kΩ with an inter-electrode impedance of ≤3 kΩ. Room lights were turned off during the collection. Participants were instructed to relax and remain still during testing.

#### 2.4.1. Frequency Following Response (FFR)

FFR responses were evoked using a 40 ms/da/stimulus generated by the IHS SmartEP advanced research module. This stimulus contains a release burst and voiced formant transition with an F0 that linearly rises from 103 to 125 Hz, with voicing beginning at 5 ms and an onset release burst during the first 10 ms. Although the stimulus does not contain a steady-state vowel, it is psychophysically perceived as a consonant–vowel speech syllable [53].

The stimulus was presented in alternating polarity to the right ear at a rate of 10.9 Hz at 85 dB nHL (equivalent to 80 dB SPL RMS) through a gold-foil TIPtrode attached to an ER-3C insert earphone. The non-test ear was unobstructed. Evoked responses were processed online through IHS. Signals were amplified with a 10^5^ gain and band-pass filtered between 100 and 3000 Hz. Responses were collected over a 58 ms epoch at a 50 kHz sample rate and averaged over 3000 repetitions. Two repeatable waveforms were collected and summed to obtain a grand average response (6000 sweeps total).

**Figure 2:**
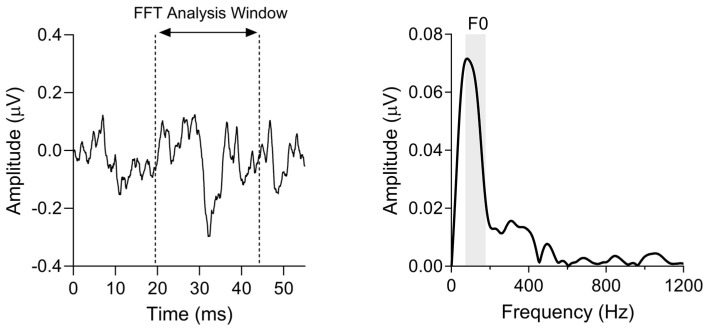
(**Left**) Representative trace of a single participant’s FFR to the 40 ms/da/stimulus in the time domain. The Fast Fourier Transform (FFT) analysis window (19.5–44.2 ms) is indicated by vertical dashed lines. (**Right**) The FFT of the same participant’s response. The shaded region represents the 100 Hz wide bin (75–175 Hz) centered on the stimulus F0 used to average the spectral magnitude (µV) of the F0 response.

Offline waveform analysis was performed using MATLAB code established in the Brainvolts Laboratory at Northwestern University. A Fast Fourier Transform was applied to the waveform from 19.5 to 44.2 ms, a region of the response corresponding to the fully voiced portion of the stimulus, omitting the unvoiced consonant release and the transient FFR component corresponding to the onset of voicing [54]. The strength of F0 encoding was defined by averaging the spectral magnitude, in µV, within a 100 Hz wide bin centered around the stimulus F0 (i.e., 75–175 Hz). F0 values outside two standard deviations of age-based normative data [55] were excluded, resulting in 65 FFR data points in the analysis. Figure 2 shows a representative FFR and its frequency analysis.

#### 2.4.2. Electrocochleography (ECochG)

ECochGs were evoked using a 100 μs broadband click, in alternating polarity, generated by the IHS SmartEP software (version 5.41.01). The stimulus was presented to the right ear at a rate of 9.1/s and 21.1/s at 88 and 84 dB nHL, respectively (equivalent to 90 dB SPL RMS), through a gold-foil TIPtrode attached to a shielded ER-3C insert earphone. The non-test ear was unobstructed. Evoked responses were processed online through the IHS Smart EP Duet platform. Signals were amplified with a 10^5^ gain and band-pass filtered between 10 and 1500 Hz. Responses were collected over a 12.8 ms epoch at a 40 kHz sample rate and averaged over 1024 repetitions. Two repeatable responses (or three for noisier responses that required more signal averaging) were added to obtain a grand average response for each click rate (total 2048 sweeps minimum, 3072 sweeps maximum).

**Figure 3:**
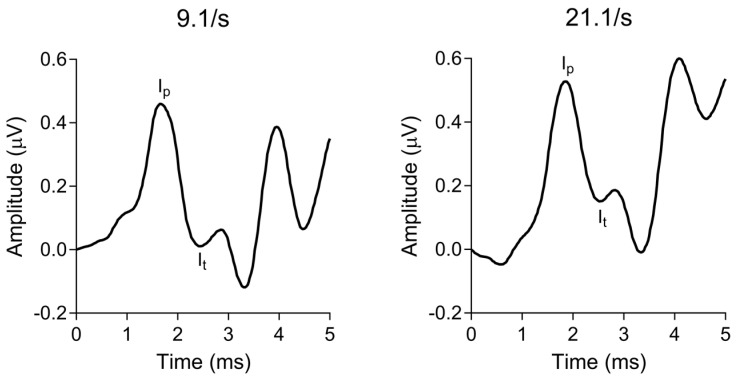
Representative ECochG responses from a single participant to broadband clicks presented at (**left**) 9.1/s and (**right**) 21.1/s. Amplitude was defined as the µV difference between wave I peak and its following trough, marked as I_p_ and I_t_, respectively. The percent change in amplitude as a function of increasing click rate was calculated for each participant and used for analysis.

Waveform components (i.e., wave I peak and following trough) were marked in the IHS SmartEP software using visual overlay cursors. All markings were identified and confirmed through a consensus between the two authors. The change in wave I amplitude as a function of increasing the click rate from 9.1/s to 21.1/s was calculated as a percentage for each participant. Two participant responses to 21.1/s were unusable (i.e., too noisy to identify waveform components reliably) and therefore excluded, resulting in 74 ECochG data points in the analysis. Figure 3 shows a representative ECochG response for each click rate presentation.

### 2.5. Statistical Analyses

The relationship between temporal processing and speech-in-noise perception was first analyzed on a performance continuum using correlational analyses. Partial correlations were performed between each measure of temporal processing and the two speech-in-noise measures (AzBio and SR2-SRM), controlling for hearing sensitivity in the standard (4-PTA) and extended (EHF-PTA) frequencies. Spearman’s rank correlations (denoted by r_s_) were run instead of Pearson’s correlations for test variables that did not pass the Shapiro–Wilk test of normality. Next, the top and bottom quartile performers (i.e., 75th and 25th percentiles, respectively) on each speech-in-noise measure were compared across measures of temporal processing using unpaired *t*-tests or Mann–Whitney U tests for non-parametric test variables. Effect sizes and confidence intervals were performed in R (version 4.3.3) using the effectsize package. The effect sizes were calculated using Hedges’ g for normally distributed data, and Cliff’s delta (δ) for non-parametric data [56]. The correlation analyses were performed in R using the ppcor package, and the group comparisons were performed in Prism (version 10.1.1; GraphPad Software, La Jolla, CA, USA).

## 3. Results

Descriptive statistics for all measures included in the analysis are listed in Table 1.

### 3.1. Peripheral Auditory Nerve Function

**Figure 4:**
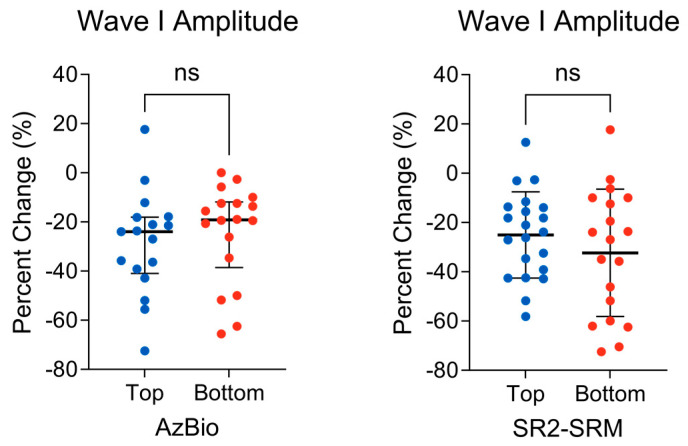
(**Left**) Distribution of the wave I amplitude change in the top (*n* = 17) and bottom (*n* = 18) AzBio performers. The bold horizontal line represents group medians, bracketed by the interquartile range. A Mann–Whitney U test revealed no significant differences between groups. (**Right**) Distribution of the wave I amplitude change in the top (*n* = 21) and bottom (*n* = 19) percentile SR2-SRM scores. The bold horizontal line represents the group means, bracketed by 1 standard deviation. An unpaired *t*-test revealed no significant difference between the groups. ns = non−significant.

Pearson’s partial correlation was computed to assess the relationship between the wave I peak-to-trough amplitude change as a function of increasing stimulus rate (i.e., peripheral auditory nerve function) and the AzBio performance while controlling for 4-PTA and EHF-PTA. No significant correlation between the two variables was found (r(70) = −0.04, *p* = 0.71). Next, a Mann–Whitney U test was conducted to determine whether there was a difference in the wave I amplitude change between the top 75th percentile (*n* = 17) and bottom 25th percentile (*n* = 18) AzBio performers. There was no difference observed between the top (mdn = −24.00) and bottom (mdn = −19.14) groups (U = 117; *p* = 0.24) (Figure 4, left). The effect size, measured by Cliff’s delta, was δ = −0.24, 95% CI [−0.56, 0.15], indicating a small effect.

Pearson’s partial correlation was computed to assess the relationship between the wave I peak-to-trough amplitude change as a function of increasing stimulus rate and SR2-SRM while controlling for 4-PTA and EHF-PTA. No significant correlation between the two variables was found (r(74) = 0.12, *p* = 0.31). Next, an unpaired *t*-test was conducted to determine whether there was a difference in the wave I amplitude change between the top (*n* = 21) and bottom (*n* = 19) percentile SR2-SRM scores. There was no difference observed between the top (M = −25.06, SD = 17.49) and bottom (M = −32.31, SD = 25.83) groups, t(38) = 1.04, *p* = 0.30 (Figure 4, right). The effect size, measured by Hedges’ g, was g = 0.33, 95% CI [−0.29, 0.94], indicating a small effect.

### 3.2. Subcortical F0 Encoding

**Figure 5:**
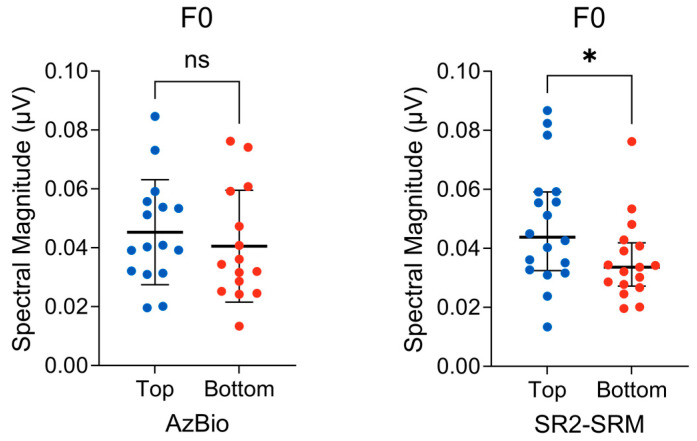
(**Left**) Distribution of the FFR F0 response magnitude in the top (*n* = 16) and bottom (*n* = 15) AzBio performers. The bold horizontal line represents the group means bracketed by 1 standard deviation. An unpaired *t*-test revealed no significant difference between the groups. (**Right**) Distribution of the FFR F0 response magnitude in the top (*n* = 18) and bottom (*n* = 17) percentile SR2-SRM scores. A Mann−Whitney U test revealed significantly lower F0 magnitudes in the bottom SR2-SRM group. The bold horizontal line represents group medians, bracketed by the interquartile range. ns = non−significant, * *p* < 0.05.

Spearman’s rank partial correlation was computed to assess the relationship between FFR F0 response magnitude (i.e., subcortical F0 encoding) and AzBio performance while controlling for 4-PTA and EHF-PTA. No significant correlation between the two variables was found (r_s_(61) = 0.11, *p* = 0.40). Next, an unpaired *t*-test was conducted to determine whether there was a difference in F0 response magnitude between the top and bottom percentile AzBio performers. There was no difference observed between the top (M = 0.045, SD = 0.017) and bottom (M = 0.040, SD = 0.018) groups, t(29) = 0.71, *p* = 0.48 (Figure 5, left). The effect size, g = 0.25, 95% CI [−0.44, 0.94], indicates a small effect.

Spearman’s rank partial correlation was computed to assess the relationship between the FFR F0 response magnitude and SR2-SRM while controlling for 4-PTA and EHF-PTA. No significant correlation between the two variables was found (r_s_(63) = 0.14, *p* = 0.24). Next, a Mann–Whitney U test was conducted to determine whether there was a difference in the F0 response magnitude between the top 75th percentile (*n* = 18) and bottom 25th percentile (*n* = 17) SR2-SRM scores. A significant difference was observed between the top (mdn = 0.043) and bottom (mdn = 0.033) groups (U = 91; *p* = 0.041) (Figure 5, right). The effect size, δ = 0.41, 95% CI [0.04, 0.67], indicates a medium effect.

### 3.3. Binaural TFS Sensitivity

**Figure 6:**
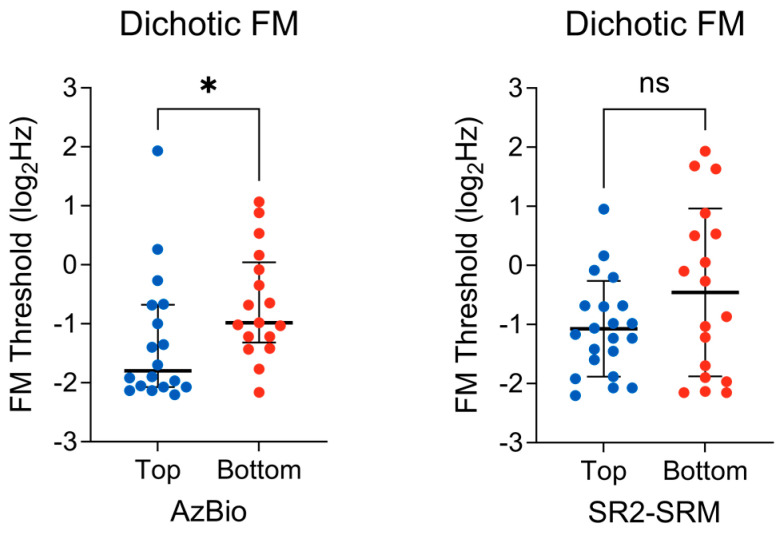
(**Left**) Distribution of the dichotic FM thresholds in the top (*n* = 18) and bottom (*n* = 17) AzBio performers. The bold horizontal line represents the group medians, bracketed by the interquartile range. A Mann–Whitney U test revealed significantly lower FM thresholds in the top AzBio performers. (**Right**) Distribution of dichotic FM thresholds in the top (*n* = 21) and bottom (*n* = 18) quarter percentile SR2-SRM scores. The bold horizontal line represents the group means, bracketed by 1 standard deviation. An unpaired *t*-test revealed no significant difference between the groups. ns = non–significant, * *p* < 0.05.

Spearman’s rank partial correlation was computed to assess the relationship between the dichotic FM detection thresholds (i.e., binaural TFS sensitivity) and AzBio performance while controlling for 4-PTA and EHF-PTA. There was a statistically significant, moderate, negative correlation between the two variables (r_s_(69) = −0.36, *p* < 0.01), showing that the lower dichotic FM thresholds (i.e., better binaural TFS sensitivity) were associated with an increase/improvement in the AzBio performance. Zero-order correlations showed a statistically significant moderate correlation (r_s_(70) = −0.33, *p* < 0.01), indicating that hearing sensitivity across standard and extended frequencies had very little influence in controlling for the relationship between dichotic FM thresholds and AzBio performance. Next, a Mann–Whitney U test was conducted to determine whether there was a difference in dichotic FM thresholds between the top 75th percentile (*n* = 18) and the bottom 25th percentile (*n* = 17) AzBio performers. There was a significant difference between the top (mdn = −1.79) and bottom (mdn = −0.98) groups (U = 88.5; *p* = 0.032) (Figure 6, left), indicating that those who performed well on the AzBio task had better binaural TFS sensitivity. The effect size, δ = −0.42, 95% CI [−0.68, −0.06], indicates a medium effect.

Spearman’s rank partial correlation was computed to assess the relationship between the dichotic FM detection thresholds and SR2-SRM while controlling for 4-PTA and EHF-PTA. No significant correlation between the two variables was found (r_s_(71) = −0.09, *p* = 0.45). Next, an unpaired *t*-test was conducted to determine whether there was a difference in the dichotic FM thresholds between the top (*n* = 21) and bottom (*n* = 18) percentile SR2-SRM scores. There was no difference observed between the top (M = −1.07, SD = 0.80) and bottom (M = −0.45, SD = 1.42) groups, t(37) = 1.68, *p* = 0.10 (Figure 6, right). The effect size, g = −0.53, 95% CI [−1.16, 0.10], indicates a medium effect.

## 4. Discussion

This study evaluated the role of auditory temporal processing in middle-aged listeners’ ability to understand speech-in-noise. Despite the popularity of this topic, few studies have specifically focused on middle-aged listeners—the most prevalent age group within this population of patients [2,13]. Thus, we aimed to study this phenomenon in the age group it first impacts. This fills a gap in the literature and provides an opportunity to explore this research question while minimizing confounds observed in older age, such as hearing loss. Three measures of temporal processing were investigated: the peripheral auditory nerve integrity, the subcortical encoding of periodic speech cues (i.e., F0), and the binaural sensitivity to TFS cues. Subcortical F0 encoding and binaural TFS sensitivity contributed to speech-in-noise understanding in different contexts. Specifically, weaker F0 encoding related to speech-in-noise deficits in the SR2 task where the target and maskers were highly perceptually similar, thus increasing the importance of fine sensory details to differentiate them. Conversely, poor TFS sensitivity related to speech-in-noise deficits in the AzBio task, where the target and masker are less perceptually similar, and fine sensory details are not as critical.

Subcortical F0 encoding significantly differed between the top and bottom quartile performers on the SR2 task. These findings suggest that listeners with stronger subcortical F0 encoding fidelity, leading to more robust representations of pitch cues, can better separate and identify a target from competing talkers. The fact that this relationship is observed in the SR2 task and not the AzBio task is unsurprising, as the SR2 task minimizes context effects and maximizes the reliance on sensory cues (e.g., pitch and spatial cues), while the AzBio task engages a mixture of bottom-up and top-down resources. As the perceptual similarity between target and maskers (i.e., informational masking) declines from the SR2 to the AzBio task, subtle deficits in pitch encoding may not be as detrimental to speech-in-noise abilities. Only when the target and competing maskers have similar acoustic features (e.g., slight F0 differences across the three SR2 talkers) will pitch encoding differences result in impaired performance. Lastly, while F0 encoding did not significantly relate to SR2 performance when analyzed on a continuum (i.e., correlational analysis), it would be worth repeating this experiment using a longer /da/stimulus, such as the 170 ms/da/ used in the previous literature comparing F0 encoding to speech-in-noise perception [8,31,33]. A longer stimulus that presents more F0 cycles [53] would likely produce a more sensitive measure of F0 encoding that could better identify subtle differences in subcortical temporal coding fidelity.

Across our continuous and group analyses, we observed a significant association between binaural TFS sensitivity and AzBio performance, such that listeners with good TFS sensitivity performed better on the AzBio task than listeners with poor TFS sensitivity. This finding is consistent with the psychoacoustic literature demonstrating the importance of TFS cues for understanding speech in background noise [7,36,37]. While it was perhaps unexpected for binaural TFS sensitivity to relate to AzBio performance over the SR2, which involved binaural judgment (i.e., spatial cues), the reduction in TFS sensitivity captured here may reflect deficits beyond sound source localization, such as a reduced ability to “listen in the dips” of fluctuating background noise [7]. The fact that TFS sensitivity relates to speech-in-noise perception in one speech-in-noise measure and not the other reiterates our earlier discussion regarding the differential reliance of temporal cues based on task characteristics. Specifically, we can postulate that TFS sensitivity may be more utilized in scenarios with less informational masking than other temporal cues. For example, when the target and masker are more perceptually distinct, performance may not critically rely on subtle differences in F0 encoding. Rather, differences in the ability to use TFS cues to “listen in the dips” would become more important. Further experiments would be required to determine the boundaries of this effect, as it likely plateaus or diminishes as the number of talkers in the masker increases and more temporal and spectral gaps are filled in. These considerations are by no means trivial when studying speech-in-noise perception, as difficulty understanding speech-in-noise spans a variety of contexts. It is equally important to keep this in mind when interpreting results across studies that use various speech-in-noise measures, all with unique talker and masker characteristics and, therefore, different sensory and cognitive demands.

Lastly, the single measure of temporal processing that did not relate to either measure of speech-in-noise perception was that of peripheral auditory nerve function. The results of this study are in line with other works [57,58,59], suggesting any possible damage occurring at the cochlear synapse/peripheral auditory nerve either cannot be reliably measured in humans or does not overtly contribute to degraded speech-in-noise perception—at least not in the cohort of middle-aged participants included in this study, who were not recruited based on risk factors associated with cochlear synaptopathy, such as excessive noise exposure [27]. Perhaps this result is due to methodological factors, such as using a stimulus paradigm that was not sensitive enough to this type of peripheral damage [60]. It is also worth considering that the wave I peak-to-trough amplitude may not be the most effective metric for identifying damage or dysfunction of these peripheral structures. Indeed, studies using alternative metrics such as the phase-locking value [61], an isolated metric of auditory nerve fiber activity via high-pass filtering [62], and a physiological measure of neural TFS phase-locking [2], provide an encouraging direction for characterizing the role of auditory nerve’s function in speech-in-noise abilities.

It should be noted that the study participants were not recruited based on a certain criterion for perceived/experienced speech-in-noise difficulties. Rather, participants across a continuum of perceived and demonstrated speech-in-noise abilities were recruited. While this recruitment approach is common in this area of study, it may not provide as rigorous an investigation into the population of patients as this research is driven by. More compelling observations might be made by comparing a group of normal-hearing listeners who both report and demonstrate speech-in-noise difficulties and a true control group (i.e., those who do not experience nor demonstrate speech-in-noise difficulties). These groups could be defined using cut-off values from normative data for both subjective (i.e., questionnaire) and behavioral measures of speech-in-noise difficulties; such has been provided for the Speech, Spatial, and Qualities of Hearing Scale [63] and Quick Speech-in-Noise test [64]. Future research investigating factors contributing to speech-in-noise difficulties in normal-hearing listeners should heed these considerations in their study design, if possible.

## 5. Conclusions

The study supports the hypothesis that temporal coding deficits contribute to differences in speech-in-noise perception in middle-aged listeners with normal hearing sensitivity. Speech-in-noise perception in middle-aged, normal-hearing listeners was associated with differences in subcortical F0 encoding and binaural TFS sensitivity, but in different contexts. Weaker subcortical F0 encoding was related to speech-in-noise deficits when perceptual similarity between the target and maskers (i.e., informational masking) was high and finer sensory cues were the primary source of distinction. Alternatively, a reduction in sensitivity to TFS cues was related to speech-in-noise deficits against a multi-talker babble that introduced relatively less informational masking and more energetic masking, suggesting that the ability to “listen in the dips” is more utilized in these listening environments than those with more acute sensory demands. While the fact that different cues are engaged in different listening conditions is rather intuitive and well-established, these results highlight the importance of carefully considering these factors for appropriate study designs and when drawing conclusions across studies.

## Figures and Tables

**Table 1:** Descriptive statistics of all measures included in the analysis.

Variable	Units	*n*	Mean (Std. Dev.)	Median	Range
4-PTA	dB HL	76	8.47 (4.68)	8.12	0–20.63
EHF-PTA	dB HL	75	21.84 (16.35)	21.25	−6.87–56.88
AzBio	Percent correct	74	66.01 (9.83)	65.84	42.11–84.78
SR2-SRM	dB benefit	76	6.51 (3.64)	6.19	−1.04–14.45
Wave I amplitude	Percent change	74	−24.84 (21.44)	−23.75	−72.5–23.81
F0 magnitude	µV	65	0.043 (0.01)	0.039	0.013–0.086
Dichotic FM threshold	Log_2_ (Hz)	74	−0.92 (1.08)	−1.19	−2.43–1.93

## Data Availability

The data presented in this study are available on request from the corresponding author.
